# Roles of Nitric Oxide in the Regulation of Reproduction: A Review

**DOI:** 10.3389/fendo.2021.752410

**Published:** 2021-11-19

**Authors:** Yuxin Luo, Yanbin Zhu, Wangdui Basang, Xin Wang, Chunjin Li, Xu Zhou

**Affiliations:** ^1^ College of Animal Science, Jilin University, Changchun, China; ^2^ State Key Laboratory of Hulless Barley and Yak Germplasm Resources and Genetic Improvement, Lhasa, China

**Keywords:** nitric oxide, reproduction, animals, female, male

## Abstract

Nitric oxide (NO) has attracted significant attention as a stellar molecule. Presently, the study of NO has penetrated every field of life science, and NO is widely distributed in various tissues and organs. This review demonstrates the importance of NO in both male and female reproductive processes in numerous ways, such as in neuromodulation, follicular and oocyte maturation, ovulation, corpus luteum degeneration, fertilization, implantation, pregnancy maintenance, labor and menstrual cycle regulation, spermatogenesis, sperm maturation, and reproduction. However, the mechanism of action of some NO is still unknown, and understanding its mechanism may contribute to the clinical treatment of some reproductive diseases.

## 1 Overview of Nitric Oxide

Before the last century, NO was considered a simple inorganic molecule that is toxic to living organisms. In 1980, Furchgott and Lawadzki found that vascular endothelial cells can produce and release a vasodilating substance, an endothelial cell-derived relaxing factor. In 1987, studies by Palmer and others showed that NO is the vasodilating substance released by vascular endothelial cells, which can lower blood pressure, inhibit vascular smooth muscle cell proliferation, and inhibit platelet adhesion (1). NO has received increasing attention in various fields of life science and has become a research hotspot in biomedicine. NO was named the “star molecule” by American Science magazine in 1992, and then the Nobel Prize in Physiology or Medicine in 1998 was awarded to three American pharmacologists for the discovery of “NO as a signal molecule of the cardiovascular system,” which aroused great attention in the field of life science toward its biological effects and mechanism of action (2).

NO is a cellular messenger and effector molecule. It has the characteristics of simple structure, easy diffusion, strong reactivity, lively and unstable properties, and short biological half-life (about 2 s) (3). Further, NO—an inorganic-free radical with dual functions in organisms—is a special biological signal transmission molecule, which is produced and released by vascular endothelial cells and participates in various physiological and pathological processes in the nervous, circulatory, respiratory, digestive, and immune systems (4). In the female reproductive system, NO influences successive reproductive processes, such as follicular development, oocyte maturation, ovulation, corpus luteum degeneration, fertilization, embryo implantation, maintenance of pregnancy, regulation of delivery, and menstrual/estrous cycle regulation. In the male reproductive system, NO affects progressive reproductive processes, such as spermatogenesis, sperm maturation, and capacitation (5). Recently, the study of NO in animal reproduction has stimulated widespread interest. Through research on the effect of NO on the reproduction of mice, pigs, cattle, and other animals, the understanding of NO has also been greatly enhanced.

### 1.1 Properties and Synthesis of NO

NO has physiological functions, such as relaxing blood vessels, transmitting nerve signals, and inhibiting platelet adhesion (6). With free radical chemical properties and diverse biological functions, it is an important information and effector molecule in organisms. It significantly affects cell information transmission, cell defense and damage, and mammalian reproductive activity (7). It is a gas signal molecule, which can act not only on the cells producing it, but also on the cells adjacent to it and widely influences various physiological and pathological processes in organisms.

*In vivo*, NO is synthesized by three kinds of nitric oxide synthase (nitric oxide synthase, NOS) (8). NOS is the rate-limiting enzyme in the NO reaction pathway. The reaction of NO *in vivo*: L-arginine + 3/2 NADPH +H^+^+ 2 O_2_= citrulline + nitric oxide + 3/2 NADP, is catalyzed by NOS (9). Three types of NOS exist: nNOS, eNOS, and iNOS (10). Among them, nNOS and eNOS are calcium-dependent, also known as cNOS, with no interspecific specificity, whereas iNOS is non-calcium-dependent with interspecific specificity. eNOS is mainly distributed in vascular endothelial cells, platelets, myocardial intima, brain, and nerve tissues of animals. Normally, it catalyzes the production of basic NO. nNOS is mainly present in the brain, spinal cord, peripheral nerves, and to a small degree, in the bronchi, trachea, and bones (11). In nerve tissues, nNOS only secretes a little NO when neurons need it. iNOS is distributed in many other tissues, except nerve tissue, and is induced by inflammation and immune response only (12). The *CNOS* gene is expressed at a low level under physiological conditions, but its activity increases rapidly under the action of agonists; the effect time occurs within a few seconds, whereas iNOS is not expressed under physiological conditions; its expression is regulated by cytokines, such as endotoxin, inflammatory cytokines, and nuclear factors, but the amount of NO produced by iNOS expression is significantly higher than that by cNOS expression; it also performs the greatest function in the cytotoxicity, cell growth inhibition, and cell protection processes (13).

### 1.2 Physiological and Pathological Effects of NO

The presence of NO in many physiological and pathological events enhances the in-depth study of NO, it may have positive or negative effects on a specific physiological or pathological event. Physiological concentrations of NO are involved in information transmission and cytoprotection. Excessive or insufficient levels can cause pathological effects. NO deficiency can be treated by supplementation, while excessive NO has a toxic effect. When the NO content increases to a level of 20 μM, it can react quickly with the surrounding superoxide anion (O_2_^-^) to form peroxynitrite (ONOO-) (14). ONOO^-^ is a strong oxidant that can effectively oxidize protein sulfhydryl groups, iron-sulfur centers, and zinc finger structures, nitrifying protein tyrosine residues, inactivating many important proteins and enzymes, affecting cell metabolism, inhibiting respiratory chain enzymes, destroying mitochondrial structure, breaking DNA, and initiating lipid peroxidation, resulting in tissue damage (15). When NO and O_2_^-^ are relatively excessive, N_2_O_3_ can be formed through a series of reactions and can react with sulfhydryl and amino groups to form nitrosamines, which can inhibit the functions of some proteins, such as the ability to repair DNA damage, resulting in cell death or gene mutation. NO, as a signal molecule or free radical, bidirectionally influences many aspects of an organism’s physiology, including apoptosis, protection from degenerative changes of the nervous system, protection from toxicity, and inhibition or promotion of a pathological process.

### 1.3 The Mechanism of NO Action

**Figure 1:**
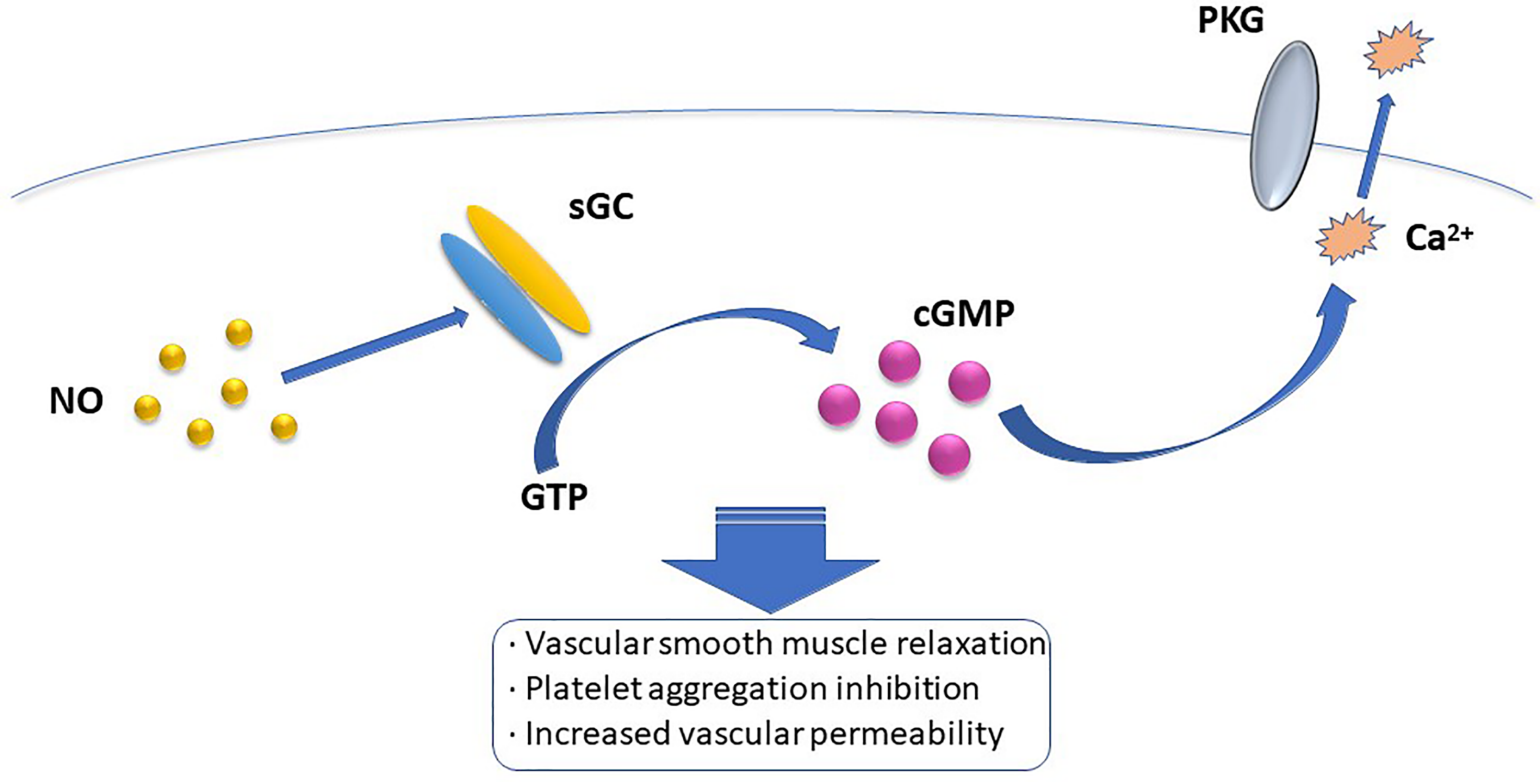
The most widely accepted mehanism of intracellular NO pathway is that NO binds to soluble guanylate cyclase (sGC) and activates sGC to increase intracellular cGMP concentration. When the concentration of cGMP increases, the activation of cGMP dependent protein kinase G induces a decrease in intracellular calcium concentration, which mediates the biological effects of vascular smooth muscle relation, platelet aggregation inhibition, and increased vascular permeability.

The biological mode of action of NO involves NO-mediated activation or inhibition of a particular enzyme, or reacting with O_2_^-^ to ONOO^-^ and cause tissue damage (16). Presently, the most widely accepted mechanism of the NO intracellular pathway is the binding of NO to soluble guanylate cyclase (sGC) and activating sGC to increase cellular cGMP concentration. When the concentration of cGMP increases, the activation of cGMP-dependent protein kinase G induces a decrease in intracellular calcium concentration, which mediates vascular smooth muscle relaxation, platelet aggregation inhibition, vascular permeability increase, and other biological effects (17) (**Figure 1**). Among the many types of GC, only sGC is the target enzyme of NO. NO can recognize Fe^2+^ and the heme-assisted group in sGC and change the pathway of sGC, thus activating the enzyme and promoting GTP cyclization to cGMP (18). However, other mechanisms of action of NO, such as affecting protein function, may be S-nitrosation-mediated. The heme-containing group reaction between NO and protein is reversible, whereas most of the effects induced by NO are irreversible, and can also be mediated by tyrosine nitration, cyclooxygenase and protein kinase C (PKC) activation, adenylate cyclooxygenase activity inhibition, and the apoptotic pathway. Besides, NO can affect intercellular and intracellular signaling pathways by inhibiting free amino acid, polypeptide, and protein additions, and activating coxidase, PKC, and apoptosis pathways (19).

## 2 Regulation of Neuroendocrine by NO

The nervous system controls NO levels as a regulator of the nitrogen energy system (20). NO exists in the hypothalamus, pituitary gland, and gonadal glands and can act on the hypothalamus to regulate gonadotropin-releasing hormone (GnRH). The position of NO-producing neurons in the hypothalamus is similar to that of GnRH neurons. NO stimulates hypothalamic neurons to secrete GnRH (21). NO can also regulate the release of gonadotropins and stimulate the formation of the preovulatory luteinizing hormone (LH) peak (22). The increase in iNOS during follicular development is consistent with the peaking of LH (23). However, some researchers believe that NO can inhibit hormonal release from pituitary cells. NO can also regulate gonadal hormones, and sex hormones and NO can regulate each other. For example, in the follicular stage, estrogen can upregulate NO levels, progesterone can downregulate NO secretion, and estradiol (E_2_) can protect blood vessels *via* NO (24). NO also influences signal transduction pathways in releasing adrenocorticosterone. In physiological and pathological processes, NO influences the neuroendocrine and neuroimmune systems and produces various interactions. nNOS affects learning regulation and memory and is involved in neuropsychiatric disorders, including depression. NO produced in stress response has been linked to behaviors similar to depression and anxiety. Besides, NO, produced by nNOS and iNOS in brain structures, is involved in regulating the HPA axis. Like neurotransmitters, NO can spread rapidly in the mammalian central nervous system membrane, which is one of the reasons why NO is more effective than other transmitters. Studies have found that when exposed to stress, the interleukin levels in the body will increase, and interleukin-related inflammation will cause iNOS expression by stress media, such as toxic fumes or chemicals, thus increasing the NO content. Therefore, iNOS and NO are related to the pathophysiology of stress-induced inflammation (25). Studies on sympathetic nerve-mediated cardiovascular diseases have found that the expression of nNOS in the paraventricular nucleus of the hypothalamus is decreased, whereas the expression of nNOS is increased in mice with spontaneous hypertension and vascular hypertension, which suggests that the expression of nNOS depends on whether the sympathetic nerve is excited (stimulated) or not (26).

## 3 The Role of NO in the Reproduction of Female Animals

### 3.1 NOS Expression in Follicles

NOS is the only enzyme involved in generating NO. Studying the distribution of NOS is of great significance in clarifying the mechanism of NO. For several mammals, NO affects ovarian steroid and follicular development, atresia, ovulation, oocyte quality, and luteal function. The three NOS subtypes were expressed in the cervix of rats, but only eNOS and iNOS were expressed in the uterus (27). Recent studies have reported the presence of nNOS in pig ovaries (28). iNOS was mainly found in the granulosa cells of healthy immature follicles in rat ovaries, while eNOS was detected in granulosa cells at all stages of follicular formation in cattle (29). NOS is expressed differently in different animals, mainly in immature follicles in mice (30), and mainly in preovulatory and luteinized follicles in pigs. eNOS was expressed in oocytes, vascular endothelial cells, granulosa cells, theca cells, and cumulus cells of porcine large follicles (7–10 mm), but not in cumulus cells of medium-sized follicles (3–6 mm) (31). In the early stage of porcine follicular development, NOS is rarely expressed in granulosa and theca cells, and nNOS and iNOS are expressed in granulosa cells with cavity follicles (32). Previous studies have found that NO/sGC/cGMP (a signaling pathway mediated by cyclic nucleotides in NO) has a functional significance in the ovary of perch and can regulate maturing follicular oocytes in bass (33). Additionally, it was found that the expression of eNOS, iNOS, and nNOS, such as in freshwater catfish, was seasonally dependent and cell-specific. All NOS subtypes are expressed only in the reproducible ovaries and not in the stationary ovaries, although very weak nNOS immunoprecipitation was occasionally found in the nuclei of perinuclear oocytes and the cytoplasm and nuclei of inactive ovarian oocytes (34). In human and pig follicles, the higher the concentration of nitrite/nitrate (NOx), the larger the follicle and the higher the concentration of E_2_ (35).

### 3.2 NO and the Growth and Development of Preantral Follicles

Follicular development is regulated by various factors, such as cytokines, growth factors, and locally produced hormonal substances, among which NO may play an important role. Studies have shown that the level of NO changes during follicular growth (36), indicating that NO may influence the process of follicular development. The presence of iNOS and eNOS in theca cells is related to the synthesis of NO and importantly affects the normal development of follicles cultured *in vitro* (37). Immature follicle development induced by chorionic gonadotropin in equine animals is related to the increased expression of eNOS, while the induction of human chorionic gonadotropin increases both iNOS and eNOS isozymes (38). In a study of preantral follicles, it was found that a 0.1 mM single-nucleotide polymorphism (SNP) did not affect antral follicle formation, but high concentrations inhibited follicular cavity formation. NO has been suggested to play a dual role in regulating follicular development. The effect of NO on follicular growth and development may be caused by vasodilation, which affects follicular blood flow (39). In the ovaries, the expression and activity of NOS subtypes vary greatly among different animal species and different ovarian processes. The expression of NOS subtypes (nNOS, iNOS, and eNOS) differs at different stages of buffalo follicle development (preantral follicles, antral follicles, and ovulating follicles). iNOS showed stronger immune reactivity in granulosa cells of these three follicle types, indicating that iNOS protein was more common than eNOS and nNOS in buffalo ovaries (40).

NO is related to the synthesis and secretion of GnRH, FSH, LH, E_2_, progesterone (P_4_), and prostaglandin in many animals (41, 42). Gonadotropins can promote the maturation of oocytes and regulate the secretion of steroid hormones in granulosa cells, thus affecting follicular development. The development of preantral follicles is the process by which follicular cells acquire the ability to respond to gonadotropins. Thyroid hormones importantly affect the reproductive function. T3 is an iodinated tyrosine converted by thyroid hormone, which can promote follicular growth and granulosa cell development induced by FSH *in vitro*. Studies have found that the synergism of T3 and FSH can significantly increase the expression and translocation of glucose transporters in cells and glucose uptake. These changes were accompanied by upregulated NOS3 expression and increased total amount of NO and activity of NOS3 (43).

### 3.3 NO and Oocyte Maturation

Oocyte maturation requires specific genes to be accurately expressed at a specific time. Concerning the effect of NO on oocyte maturation, it was found that the number of ovulations was reduced and meiosis was abnormal in mice injected with NOS inhibitor. Compared with that of normal mice, the ovulation rate of eNOS knockout mice was significantly reduced, oocyte meiosis was abnormal, and oocyte mortality was increased (44). It was further confirmed that NO significantly affects oocyte maturation, which is related to cumulus cells. The maturation of the cumulus-oocyte complex is controlled by the NO/NOS system (45). The results showed that aminoguanidine (AG, a specific iNOS inhibitor) significantly inhibited the recovery of meiosis in porcine oocytes, while L-NAME inhibited the excretion of the first polar body. In early follicular development, eNOS was less expressed in oocytes and granulosa cells, whereas in late follicular development, eNOS was more significantly expressed in oocytes and granulosa cells (46).

A certain concentration of NO can promote the maturation of oocytes; iNOS-derived NO can regulate the rupture of mouse germinal vesicles and the excretion of the first polar body (47). The lack of NO inhibits meiosis and maturation of oocytes. For example, the NOS inhibitor L-NAME has a significant inhibitory effect on oocyte maturation, and this inhibitory effect can be altered by SNP (45). This may be due to increased cGMP levels and decreased cAMP activation, which promote oocyte maturation. However, excessive NO also inhibits oocyte maturation, which is achieved by inhibiting E_2_ secretion from the pregnant equine granulosa cells of follicles. At present, the specific mechanism of NO/NOS in oocyte maturation is not clear and requires further study.

### 3.4 NO and ovulation

Ovulation—a complex process involving gonadotropins, steroids, some cytokines, prostaglandins, leukotrienes, and histamines—involves various ovarian cells and requires several information transmission pathways and specific gene expression. Currently, a large amount of data show that NO is related to ovulation (48). NO levels increase with follicular development (28), and this increase was associated with E_2_ concentration. NO is associated with vascular remodeling. Therefore, NO is speculated to be an important mediator of vascular changes and tissue remodeling during ovulation and luteinization. Both non-specific NOS inhibitors, AG and L-NAME, significantly inhibited ovulation in rats in a dose-dependent manner, and this inhibitory effect could be reversed by NO donor SNP (49). NO production is an important physiological feature in normal ovulation cycles. In a study of mouse follicles, it was found that a local level of NO activity was necessary for normal follicular development. NO deficiency reduced the growth and ovulation of mouse follicles. For example, L-NAME treatment in rabbits reduced the production of large follicles, *in vitro* L-NAME treatment reduced the rate of follicular rupture, and NO gene knockout mice showed altered estrous cycle and reduced ovulation rate (50). Excessive NO also inhibits ovulation, including disruption of tyrosine kinase domain of Ron receptor knockout, increasing NO levels and subsequently decreasing ovarian volume and ovulation rate (51).

NO can regulate ovulation *via* hormones. NO can increase the release of LH before ovulation, thus promoting the occurrence of a preovulatory LH peak, while LH acts on theca cells, promoting theca interna cell differentiation and androgen secretion, and stimulating follicle development, maturation, and ovulation (52). In mice, inhibin A produced by FSH-stimulated granulosa cells mediated the formation of the LH peak before ovulation (22). NO regulates many hormones, including those of the entire reproductive endocrine axis, such as E_2_, which exhibits a bidirectional regulatory effect on ovulation. For example, in mice, NO levels were consistently elevated before ovulation, while E_2_ and LH were correspondingly upregulated and peaked just before ovulation. Simultaneously, some studies have shown that NO can inhibit E_2_ synthesis in granulosa cells when increased to a certain concentration. NO inhibits steroid hormone synthesis, promotes ovulation, and inhibits differentiation *via* resting granulocyte cells (53). The main physiological mechanism by which NO inhibits E_2_ synthesis in granulosa cells is as follows: 1) NO inhibits E_2_ synthesis in mice by reducing aromatase mRNA and inhibiting aromatase activity, 2) NO inhibits nitrourea formation by binding to the cysteine of aromatase heme, and 3) the endometrium can express high mobility group box 1 (HMGB1) in a process which is regulated by E_2_, progesterone, and NO. E_2_ increases the expression of HMGB1, and progesterone decreases the expression of HMGB1. Both E_2_ and progesterone can regulate the expression of iNOS and the production of NO in the endometrium (54). After the eNOS gene was knocked out, the secretion of progesterone in the ovaries of mice was significantly reduced.

NO deficiency inhibits GnRH release and affects release of FSH and LH. For example, Barnes found that chronic NO deficiency decreased the release of GnRH in the hypothalamus of female rats, accompanied by a decrease in LH and FSH secretion. Similarly, excessive NO can also inhibit the secretion of pituitary and hypothalamic hormones. Mouse graft experiments *in vitro* have shown that excessive NO can inhibit the release of GnRH and LH (55, 56).

NO can also regulate ovulation by affecting other reproductive hormones. In mice, NO can inhibit the release of inhibin—a hormone associated with oocyte maturation and stimulated by FSH—to mediate the formation of LH peak before ovulation and enhance follicular exception by producing PGE and PGF2a *via* the trioxygenase pathway. The process of NO-facilitated hormonal ovulation is more complex and involves not only the hormone and NO regulating each other, but also many other substances.

NO can also affect vascular permeability to regulate follicular pressure and rupture. For example, NOS inhibitors can inhibit the expansion of mouse follicular vessels, reduce the flow of follicular fluid into the follicular cavity (57), reduce follicular pressure, and affect ovulation.

### 3.5 NO and Follicular Atresia

Studies have shown that cGMP is related to oocyte maturation, and intracellular cGMP can be regulated by NO. Three NO synthases and four soluble Guarnidase (sGC) were found to be expressed in the ovary. Interestingly, activation or inhibition of the NO/sGC/cGMP pathway in fully grown follicles may lead to oocyte maturation. During oocyte maturation, cGMP level in follicular cell layer increased, oocyte level decreased, NO level in follicular cells increased and oocyte remained constant (58).

The microenvironment of ovarian follicles is also critical for normal oocyte development, follicular generation, and timely ovulation. High concentrations of NO in the follicular fluid can be harmful, affecting the maturation of oocytes and embryos, and greatly reducing the success rate of *in vitro* fertilization (59, 60). However, low NO3/NO2 level in follicular fluid is beneficial to fertilization and stable embryo implantation (61). Studies have shown that NO in follicular fluid is a marker of follicular hypoxia and suboptimal embryo development (62). In addition, having endometritis reduces the level of nitric oxide in the follicular fluid, impairing the growth and composition of the largest follicles (63).

The follicles that finally ovulate in the ovary undergo follicular genesis, follicular recruitment, and follicular selection. During the selection, most follicles undergo physiologic atresia. Finally, only a few follicles develop into the dominant follicle and mature at ovulation. Normal follicular function may be reflected in cell proliferation and apoptosis, the expression of regulatory factors, and the production of hormones that essentially affect reproduction and the production of healthy offspring (64). Whether developing follicles form dominant follicles or closed follicles is dependent on many factors. NO can influence follicular atresia. Many studies have shown that follicular atresia is the result of follicular cell apoptosis; that is, apoptosis is the mechanism of follicular atresia. Follicular atresia is regulated by complex signals of cell death and survival (65).

NO mainly affects follicular atresia by affecting apoptosis in the granulosa cells. Studies have found that iNOS can inhibit the apoptosis of rat granulosa cells *via* autocrine and paracrine pathways and prevent early follicular atresia. The results showed that the spontaneous apoptosis of cultured granulosa cells from rats could be directly inhibited by SNP treatment without the addition of donor S-nitrose-N-acetylpenicillamine (29). Granulosa cell degeneration is hypothesized to be the cause of follicular degeneration. Granulosa cells often degenerate first. The inability of granulosa cells to transport nutrients and signaling factors to oocytes leads to microvillus degeneration of oocytes and oocyte atresia. NO can also inhibit apoptosis of preovulation follicles in mice by stimulating heat shock protein 70 (HSP70) and inhibiting Bax expression (66). Studies have shown that the Fas/FasL system is a key factor mediating apoptosis during ovarian follicular atresia. NO can regulate the expression of apoptosis-related genes in granulosa cells. In the detection of related proteins, NO was found to inhibit the apoptosis of human granulosa cells by reducing the expression of genes that promote granulosa cell apoptosis, such as Fas, P53, and Bax, and promoting the expression of genes that inhibit apoptosis, such as Bcl-2 and HSP70. NO can inhibit the apoptosis of granulosa cells by inhibiting the cysteine aspartic enzyme in the Fas/FasL-mediated apoptosis pathway (67). NO has a dual effect of promoting or inhibiting apoptosis. It has been found that high levels of NO can inhibit the apoptosis of bovine granulosa cells (68), while NO can promote the apoptosis of well-differentiated granulosa cells (such as macrofollicular granulosa cells). NO not only regulates cell apoptosis during follicular development but also the survival and apoptosis of follicular granulosa cells after ovulation, thus modulating the estrus cycle.

The role of NO in controlling follicular angiogenesis depends on the establishment and continuous remodeling of complex vascular systems. This enables the follicle to receive the nutrients, oxygen, and hormonal support it needs and promotes the release of steroids (69).

NO can also regulate follicular atresia through its interaction with hormones. NO chiefly regulates the secretion of estrogen and progesterone; for example, progesterone can promote follicular atresia by inhibiting the frequency of LH fluctuations; however, the mechanism remains unclear. NO may be involved in the regulation of apoptosis in the whole follicular development cycle. However, how NO regulates follicular atresia through apoptosis remains controversial, as it has been documented to exhibit a dual effect, mainly depending on the concentration.

### 3.6 NO and Fertilization

Studies have shown that NO is produced by cells in the female reproductive tract. The enzyme responsible for NO synthesis can be regulated by sex hormones, and NO levels change during the estrus cycle, thus regulating fertilization. NO can affect oocyte activation during fertilization by regulating Ca^2+^, and the NO in fertilization is derived from the activation of eNOS in spermatozoa and oocytes. The NO-mediated regulation of oocyte Ca^2+^ influences the chain regulation of several substances. The increased expression of NO first activates guanylate cyclase, and the increased cGMP activates cyclic adenosine diphosphate ribose to promote the release of Ca^2+^ under conditions of inositol triphosphate activation. During fertilization, NO may be obtained from the spermatozoa, which has abundant NOS. NO can be produced during the sperm replacement reaction at 30-45 s after fertilization; as the expression of NO increases in spermatozoa, Ca^2+^ also increases. Because nNOS is sensitive to Ca^2+^, the continuous increase in Ca^2+^ promotes the production of many NO molecules.

NO regulates sperm capacitation, acrosomal reaction, sperm motility, and may also have an antiapoptotic effect. Low concentrations of NO may physiologically affect fertilization by enhancing the ability to bind to the zona pellucida (ZP), rather than by inducing acrosomal reaction or promoting oocyte penetration. The addition of a NOS inhibitor to the capacitation medium of the human spermatozoa can reduce the fertilization rate. When spermatozoa were treated with L-NAME, they could bind the ZP but not the cytoplasmic membrane (70). The activation of NOS may require binding the cytoplasmic membrane. A low concentration of L-NAME may increase the fertilization rate of cryopreserved mouse oocytes, whereas a high concentration of L-NAME (10-3 M) or SNP (10-6 M) inhibits the normal functions of spermatozoa and oocytes, indicating that there is normal physiological demand within a certain range, which leads to toxicity if exceeded. For example, polyspermia may occur in some cases of infertility, while previous studies have focused on the role of NO in reproductive physiology; the optimal regulation of NOS enzymes is key to this process.

### 3.7 NO and Embryonic Development

Embryos develop rapidly and require messengers to transmit information quickly. As a signaling molecule, NO transmits information *via* diffusion, participates in the regulation of the division and differentiation of mammalian embryos, and importantly affects cell proliferation and differentiation. NO is required in instances where the rat embryo develops from two to four cells (71). Studies have shown that embryonic development is delayed or stemmed by inhibition mediators from the two-cell to the blastocyst stage (72). When embryos were treated with exogenous NO, it was found that 0.1 M SNP inhibited the transformation of mouse mulberry embryos into blastocysts but promoted the growth of trophoblasts, indicating that different concentrations of SNP had different effects on embryos at different developmental stages. An appropriate increase in NO significantly regulates the embryonic development, and the demand for NO may be high during the development of the embryo; a too-high or too-low NO level may interfere with normal development.

Moreover, studies have shown that the level of NO significantly affects the development of embryos during their early development (73). A high level of NO changes the position of the heart from right to left, causing situs invertus (74). Embryos may be affected by various factors during development, and damage restoration is a complex process that requires not only proteins but also RNAs. Studies have shown that NO essentially influences embryonic damage, inflammation, blood vessel formation, and is important for the expression of treatment-related genes. However, such damage mainly occurs in the early stages of embryonic development, and NO-mediated tissue remodeling can occur under the skin, but the process requires a relatively long time (75).

### 3.8 Effects of NO on Reproductive Cycle and Fertility Of Female Animals

NOS requires significantly longer time to be knocked-out, but iNOS does not, suggesting that eNOS-derived NO rather than iNOS-derived NO affects the reproductive cycle and fertility of female animals; mice that drank L-NAME water and those lacking NO continually exhibited a state of persistent estrus owing to the increase in E_2_ production (76). By measuring NOS at different stages of estrus and the menstrual cycle, different subtypes of NOS were found in the fallopian tubes of mice and humans, and increased expression of iNOS was found in the fallopian tubes of women with ectopic pregnancy, such as that involving heart inversion. The increased iNOS activity may affect ovulation, ciliary pulsing, contraction, and embryo transport, thereby increasing the risk of ectopic pregnancy. These results suggest that iNOS essentially affects the reproductive cycle and infection-mediated ectopic pregnancy. As a fungicide, NO is lethal to intracellular pathogens and causes diseases such as trachoma; therefore, it is considered a part of the innate immune response and can protectively influence embryo development (77).

### 3.9 Regulation of NO on Pregnancy Physiology

#### 3.9.1 The Role of NO in Embryo Implantation

Embryo implantation is a complex process that requires endometrial interactions. Previous studies have shown that NOS essentially affects trophoblast cell recognition and remodeling, and only appropriate concentrations can lead to successful embryo implantation (78).

NO regulates embryo implantation from two aspects of embryo development and helps create the uterine environment, and it can regulate the cAMP/cGMP ratio to affect embryo division and differentiation. The cAMP/cGMP ratio significantly affects embryo growth, development, and differentiation. NO promotes the production of cGMP, which is the main regulator of the cAMP/cGMP ratio; thus, normal embryonic development requires NO regulation (79).

NOS can be detected before and during the implantation of early mammalian embryos. Studies have shown that mouse embryos produce NO during the first four days of pregnancy. When embryos after the two-cell stage were cultured in a medium containing L-NAME, the proportion of their development to the corresponding later stage was significantly lower than that in the control group, and this block could be relieved by E_2_, because E_2_ can stimulate NO production (72). Besides, the normal implantation of embryos requires a suitable uterine environment, and NOS activity in the uterus is significantly increased before implantation, indicating that NO is necessary for the formation of the uterine microenvironment. Hindering NO production will trigger abnormal implantation (80–82). In the process of embryo implantation, the endometrium undergoes complex changes. Because NO can relax the muscle layer and vascular smooth muscle, inhibit platelet aggregation, and play a role in inflammation, it is a strong candidate molecule for mediating the roles of various steroid hormones and cytokines in the endometrium and decidua (83).

#### 3.9.2 The Role of NO in Pregnancy Maintenance

An NO system is known to exist in the uterus, mainly to relax uterine muscle cells, maintain relative rest during pregnancy, and conserve uterine-placental blood perfusion. The increase in NO production during pregnancy inhibits the spontaneous contraction of the uterine muscle bundle, causes vasodilation of the maternal system, reduces the vascular response, and affects the adaptive regulation of maternal cardiovascular system function during pregnancy. During pregnancy, the concentration of NO in the myometrium and placenta increases, which contributes to uterine calm and control of uterus-fetal-placental blood flow (4).

Besides, NO significantly influences placental function and fetal development. NO is a powerful local vascular inhibitor that essentially affects hemodynamics, helping to maintain low vascular resistance in the placental circulation. Successful pregnancy requires maintenance of sufficient placental blood flow, and the placenta lacks autonomic innervation. NO is a strong vascular relaxation factor that strengthens placental blood flow and the exchange of oxygen and nutrients by dilating the umbilical artery vessels. Insufficient production of NO in the fetus and placenta triggers fetal growth retardation. Many studies have shown that the expression of NOS (eNOS) in endothelial cells differs between normal and abnormal pregnancies, and the endocannabinoid system can regulate NO formation or release; however, long-term exposure to four hydrogen marijuana may affect the transfer of maternal and child trace elements, if marijuana use during pregnancy affects the placenta, leading to adverse pregnancy, such as preterm birth and fetal growth restriction. Under pathological conditions, the fetus may be exposed to unhealthy endocannabinoid levels, which may interfere with fetal development and lead to neurophysiological abnormalities.

#### 3.9.3 The Role of NO in the Initiation of Delivery

It was found that the changes in P_4_ before and after delivery had an important influence on the initiation of delivery. P_4_ can inhibit uterine muscle contraction and stabilize the uterus during pregnancy and, once released, is an important inducer of the initiation of delivery. Uterine NO synthesis and NOS expression are P_4_-dependent. The initiation of delivery was related to a decrease in NOS activity in the uterus. iNOS levels began to decrease in the third trimester of pregnancy, and uterine NO decreased to the lowest level at the onset of delivery. Further, NO inhibition was relieved, uterine muscle excitability increased, and delivery was initiated. Besides its effect on the uterus, NO also affects the pelvis during delivery. During delivery, the expression of NOS in the pelvis increases significantly, and the production of NO increases, which promotes the opening of the pelvis (84). Relevant studies have revealed that the coordination between endothelium-derived hyperpolarizing factor and NO may also affect gender. When endothelial cells are lacking NO, the endothelium-derived hyperpolarizing factor-mediated response compensates for the absence of NO, thus achieving gender control. Therefore, it can be speculated that endothelium-derived factors are more important in the female reproductive system than in the male reproductive system (85).

## 4 The Role of NO in Male Reproduction

### 4.1 NO and Sperm

NO seems to play a major role in the regulation of sperm motility, hyperactivation, capacitation, and fertilization (86). Sperm capacitation—a very complex physiological process in which sperm undergoes a series of physiological and morphological changes, and finally obtains the ability to fertilize—is the premise of fertilization, which is affected by many factors, including NO.

Regarding sperm capacitation, NOS has been reported to be expressed in the acrosome and tail of human and mouse spermatozoa. Some researchers hypothesized that low concentrations of NO can improve sperm capacitation. The effect of NO on sperm motility is related to its concentration. Studies have shown that low NO concentrations can promote sperm motility (85), whereas high NO concentrations can inhibit sperm motility (87). The regulatory effect of NO on sperm was not only related to the NO concentration but also to the exposure time. Other studies have shown that NO has no effect on sperm motility or acrosomal response rates. Previous studies have shown that acrylamide induces the activation and release of bovine sperm from oviduct epithelial cells and that acrylamide acts through NO. Arachidonic acid ethanolamide is an endocannabinoid receptor. Activation of the cannabinoid receptor increases NO levels and stimulates sperm capacitation or release in the fallopian tube bank (88).

In terms of motility, stimulation of NO synthesis triggered an immediate decrease in sperm motility, but only at the beginning of the incubation period. After this period, the motility was restored. When sperms were cultured without NO, it was observed that all or part of the motility characteristics of all sperms were reduced, resulting in sperm fixation. Sperm motility is closely related to ATP, and the reduced respiration of sperm also affects sperm motility. The significant inhibition of sperm motility is unlikely to be due to the inhibition of ATP activity, thus reducing the intracellular ATP concentration. Besides, studies have shown that H_2_O_2_ may be involved in this effect.

For diseases, the increased activity of NOS and the increased tyrosine nitrification may be related to the pathogenesis of idiopathic asthenospermia, and the regulation of NO regarding sperm depends not only on the concentration of NO, but also on the time of exposure, which is beneficial to sperm motility. A 1997 study showed that the addition of synthetic inhibitors of NO could effectively reduce NO toxicity to sperms. Moreover, studies have shown that leptin has a certain effect on sperm motility: leptin can influence the survival of pig sperm; leptin in the hypothalamus-pituitary level affects reproductive function and is also involved in the regulation of some peripheral functions; and leptin-treated male gametes can increase the NO content, triggering the acrosome reaction (89). NO is also associated with certain diseases. For example, in patients with varicocele, the iNOS subtype induces high NO production in varicocele, which can cause decreased sperm motility and even infertility (90).

### 4.2 Regulation of Male Reproductive Tissues by NO

NOS is widely found in Leydig cells, Sertoli cells, spermatocytes, neuronal plexus in the adventitia of arterioles, immature sperm head, vascular endothelial cells, and smooth muscle cells, indicating that NO/NOS can maintain the tension of testicular arterioles, regulate testosterone secretion, and affect sperm development. Moreover, the *in vitro* culture of interstitial cells or seminiferous tubules showed that NOS was also expressed in interstitial cells and blood vessels, indicating that the testis itself can produce NO (91). Besides, the concentration of NO can determine the sperm level. The NO-CGMP pathway is activated in testicular cells, which may participate in regulating testicular functions, such as spermatogenesis and steroid production.

Pankaj and Chandra found that injection of 5-hydroxytryptamine and L-dopamine at 8 h intervals inhibited the activity of reproductive tissues and significantly decreased the concentration of nitrate-nitrite and NO in reproductive tissues, whereas the opposite results were observed at 12 h intervals (92). However, the concentration of nitric acid-nitrite in the reproductive tissues increased after reinjection of SNP at 8 h intervals; the result was the opposite after injection of L-NAME at 12 h intervals, indicating that the lack of changes in activity *in vivo* could affect the function of reproductive tissues. Experimental studies found that NO content, NOS activity, and pathological scores of the contralateral testis in the unilateral testicular torsion group were all increased to different degrees, and NO content was positively correlated with pathological score, indicating that the higher the NO concentration, the greater the tissue injury. The injury may result from the formation of more toxic ONOO-, by the combination of NO with oxygen-free radicals on the torsional side, which may damage the contralateral testicular tissue. Alternatively, it may act on the contralateral testicular tissue by accumulating proinflammatory transmitters of NO, excessively dilating blood vessels, and inducing apoptosis (93). Simultaneously, the above-mentioned injury can induce further NOS activation to synthesize NO and cause greater damage, indicating that the effect of NO on reproductive tissues is dose-dependent.

NO can inhibit testosterone secretion. Therefore, NO may regulate the endocrine function of testosterone through a paracrine mechanism. The expression of nNOS is influenced by testosterone levels in males. In addition, the two-concentration hypothesis of NO states that low concentrations of NO promote cancer, while high concentrations prevent cancer. Although prostate cancer is a hormone-driven malignancy, research has shifted from epithelial cells that respond to androgens to focus on NO therapy, tumor microenvironment, and inflammation. NO has been reported to inhibit androgen receptor activity. This may prevent prostate growth, but low levels of NO can in turn select for anti-circumcision prostate cells, producing an aggressive cancer phenotype. At high levels, nitro stress caused by NO overproduction can prevent prostate tumors (94)

**Figure 2:**
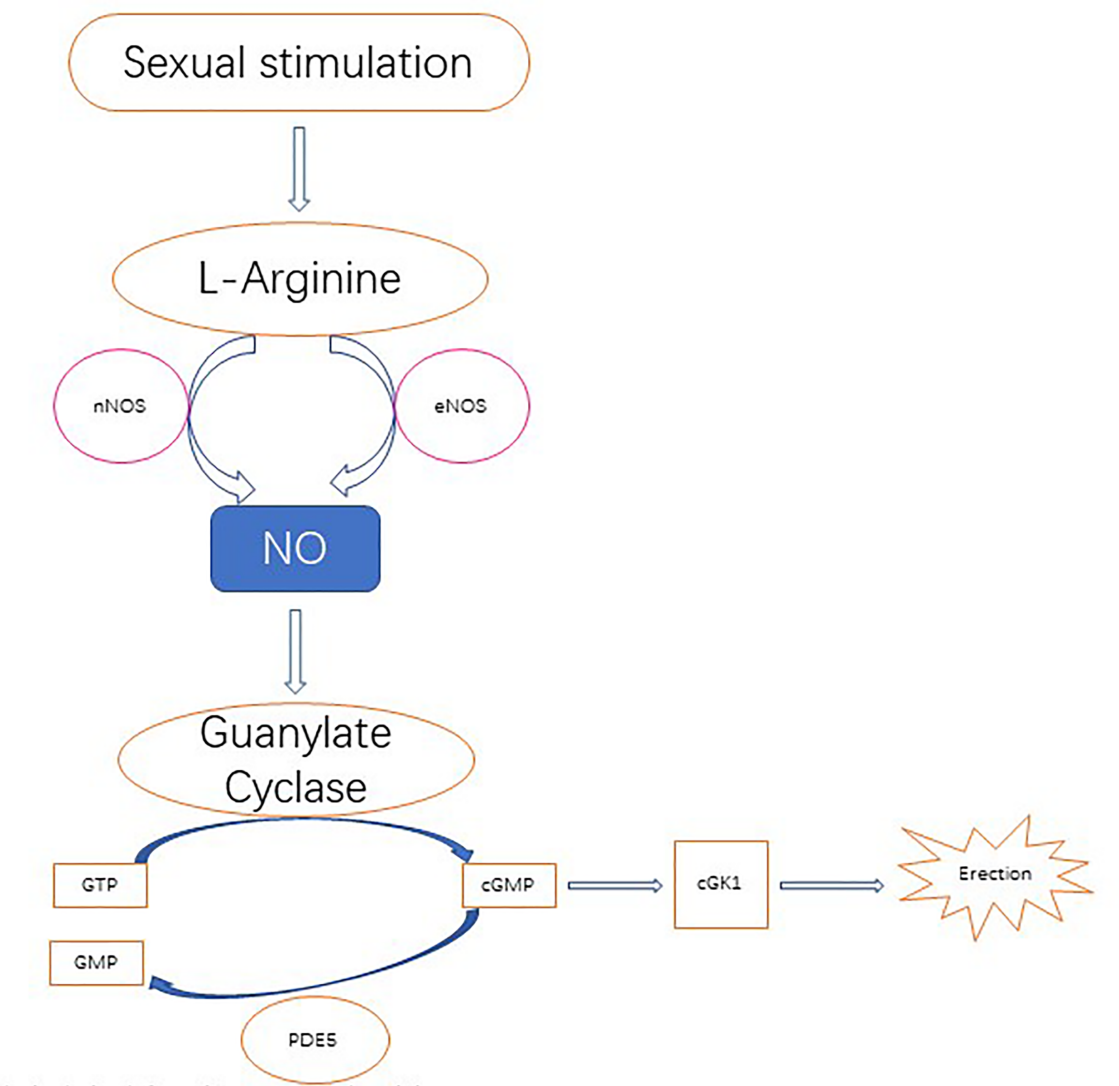
Nitric oxide is derived from its precursor l-arginine and is primarily localized to the nerves and endothelium through the catalysis of neuronal NOS (nNOS) and endothelial NOS (eNOS) following upstream stimulation, respectively. Nitric oxide diffuses to local smooth muscle cells, where it activates guanosine cyclase and then converts 5’guanosine triphosphate (GTP) to 3', 5'-cycloguanosine monophosphate (cGMP). In turn, cGMP activates cGMP specific protein kinase l (cGK l), which produces erections via downstream effectors. CGMP degrades in the catalytic action of phosphodiesterase type 5 (PDE 5) to produce inactivated nucleotide GMP. PDE 5 is a molecular target of PDE 5 inhibitor therapy, which enhances nitric oxide signaling.

Importantly, NO plays an important role in the biology of the penis, especially for penile erection. Studies have found that NO causes muscle smooth muscle relaxation, which is the basis of an erection. In this process, NO synthase subtypes have different roles. nNOS initiates erectile response, eNOS promotes maximum erectile response, and iNOS inhibition may lead to penile oxidative stress, suggesting that iNOS may actually promote the protective mechanism of fibrosis and abnormal wound healing. The nitric oxide signaling pathway regulating penile erection is shown in **Figure 2** (95).

## 5 Role of NO in Other Therapeutic Areas

NO, originally considered an environmental pollutant, has gained momentum since its discovery as an endothelial growth factor in 1987. The presence of NO in many physiological and pathological events has enhanced the further study of NO. In clinical studies, NO can be used to treat a variety of diseases: it can be used to treat cardiovascular diseases, such as coronary heart disease, acute myocardial infarction, atherosclerosis, essential hypertension, etc. (96), and can regulate vasodilation, blood pressure and blood flow in the circulatory system (97). As neurotransmitters in the nervous system, modulating the release of various neurotransmitters and mediating excitatory amino acids, synaptic transmission, and neurotoxicity (98), NOS inhibitors are used to treat stroke and reduce NO production to control epileptic seizures. It also affects the respiratory system by smooth muscle contraction and pulmonary vasoconstriction (99). Respiratory NO inhalation therapy has been used to treat persistent pulmonary hypertension, primary pulmonary hypertension in neonates, chronic obstructive pulmonary disease, adult respiratory distress syndrome and congenital heart disease with pulmonary hypertension (100). In the respiratory system, NO can be used not only as a diagnostic aid, but also as a management tool to assess severity, monitor treatment response, control asthma symptoms, and identify allergic airway inflammation (101). Because it is non-invasive and readily available, its utility in diagnosing and managing various respiratory diseases has been studied. Most studies have focused on asthma, and many support the use of NO to help diagnose asthma, predict steroid responses, and prevent worsening by directing drug dosing and assessing compliance (102). In the immune system, NO as an immunomodulatory molecule affects the cytotoxicity of macrophages (103), and the selective NOS antagonist dexamethasone is used to specifically inhibit iNOS in the treatment of endotoxin shock (104). A series of *in vitro* and *in vivo* detection methods for NO were developed. These quantitative methods will contribute to a comprehensive understanding of its biological functions and effects (105).

Therefore, a wide range of NO donors has emerged as a potential treatment for cardiovascular and respiratory diseases and cancer, promoting wound healing, and improving immune response to infection. However, short half-life, chemical reactivity, rapid systemic clearance, and cytotoxicity hinder the clinical development of most low molecular weight NO donors. Therefore, in order to control NO release, efforts are being made to design novel NO-releasing biomaterials for tumor targeting (106).

NO is an antibacterial and anti-inflammatory molecule that plays a key role in pulmonary vascular function under viral infection and other lung conditions. For example, studies have demonstrated the rationale for the use of exogenous NO to control the pathogenesis of COVID-19 and highlighted its potential to help improve clinical outcomes and relieve the rapidly rising pressures on healthcare capacity (107). NO is well known as a vascular adsorbent, an important coagulation mediator, an antimicrobial agent, and an inhibitor of SARS-COV replication. Exhaled NO is strongly associated with the type 2 inflammatory response found in asthma, and the use of inhaled NO has been an effective treatment during this pandemic, as ventilator-perfusion rates in COVID-19 patients have subsequently improved and mechanical ventilation is not required (108).

Because NO is an antibacterial and anti-inflammatory molecule, it represents a potential therapeutic agent for wounds because of its ability to regulate inflammation and eradicate bacterial infections. NO can be utilized for wound healing, with special attention to chronic wounds associated with diabetes (109). NO also plays an important role in the treatment of glaucoma, a disease that causes irreversible blindness. NO regulates blood flow in the eye and protects the optic nerve. Therefore, NO has broad research prospects for anti-glaucoma drugs (110). Studies have shown that NO is related to the aging of various plant organs; however, whether it can be applied to human medical cosmetology remains to be studied, which will be a potential development (111). On the cardiac side, inhalation of NO can be used to treat a variety of cardiopulmonary disorders, including pulmonary hypertension in children and adults and cardiac arrest syndrome (112). However, the popularity may not be very widespread for cost reasons, and efforts are currently being made to develop cost-effective and simple devices for inhalation therapy (113). In the digestive system, NO plays an important role in gastrointestinal mucosal defense and the pathogenesis of a variety of gastrointestinal diseases, such as irritable bowel syndrome and inflammatory bowel disease. The potent cellular protective effects of NO have been demonstrated in a range of animal models. However, in some disease states, inhibition of NO synthesis may be beneficial. Several attempts have been made to develop drugs to treat gastrointestinal ulcers and/or inflammatory diseases, with varying degrees of success (114). At the same time, NO also plays a role in insulin secretion and glucose metabolism, which can improve diabetes (115).

**Figure 3:**
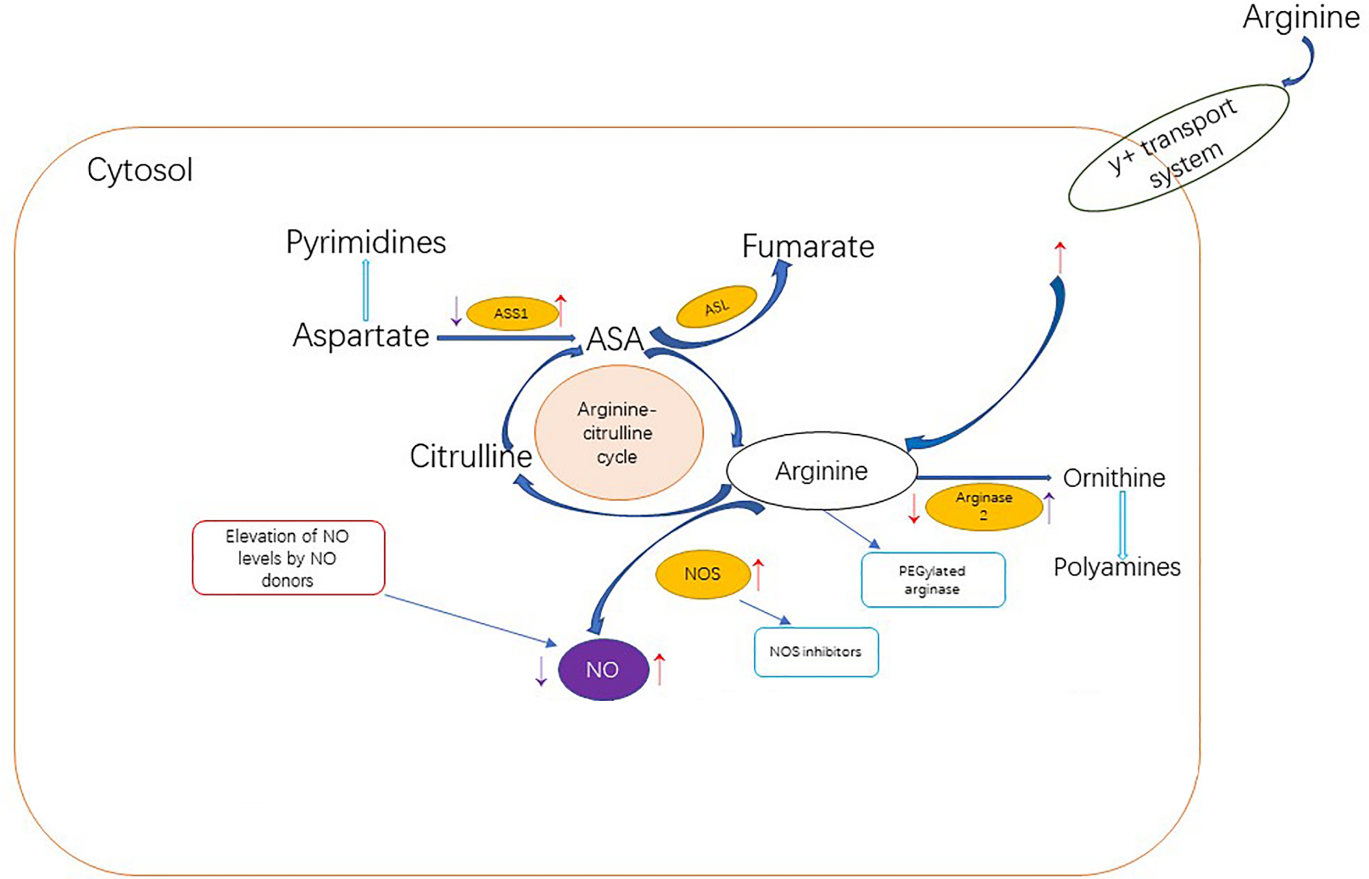
Dietary arginine can enter cells through the Y + transport system or be synthesized endogenous through the arginine-citrulline cycle.The red arrows in the figure indicate up-regulation or down-regulation of cancer-related NO metabolism proteins, resulting in a net increase in NO production. Tumor cells promote NO production through the following pathways: upregulating NOS level; Increased arginine transport;ASS1 and ASL levels were increased to improve the availability of arginine for NO synthesis. Or reduce arginine metabolism by inhibiting arginase The purple arows indicate up-regulation or down-regulation of cancer-related PROTEINS associated with NO metabolism, resulting in a net reduction in NO production. In addition to limiting NO levels, inhibition of ASS1 and upregulation of ARG2 may also support cancer metabolically by increasing pyrimidine and polyamine production. Anti-cancer NO related strategies to increase NO level are represented in the red box, while stategies to decrease NO level are represented in the blue box.

Research has shown that NO also plays a big role in cancer treatment. Low levels of NO can inhibit apoptosis and promote cancer, while high levels of NO can promote apoptosis of cancer cells. NO is an important product of arginine in cancer cells, so arginine consumption may contribute to tumor suppression by reducing the level of NO in cells. NO related anticancer strategies include those to increase NO levels and those to decrease NO levels (**Figure 3**) (116).

## 6 Conclusion

In conclusion, NO affects all stages of the mammalian reproductive process and is expressed differently at different stages. NO is involved in various physiological and pathological processes of nervous, circulatory, respiratory, digestive and immune systems and is associated with a variety of diseases. So, normal secretion of NO has some significance for many diseases. If this disorder occurs, it can lead to related diseases, such as diabetes, cardiovascular disease, pulmonary hypertension, rheumatoid arthritis, gastritis or stomach ulcers, and even cancer or tumors.

In the female reproductive system, NO affects reproductive processes such as follicular development, oocyte maturation, ovulation, luteinization, fertilization, embryo development, pregnancy maintenance, childbirth, and regulation of the menstrual cycle. At the same time, if NO is not secreted during pregnancy, it can lead to fetal abnormalities. In the male reproductive system, NO affects a series of reproductive processes such as spermatogenesis, sperm maturation, and capacitation. Serious deficiency will cause a decline sperm vitality or even infertility. All these indicate the importance of NO in living organisms. However, the mechanisms by which NO mediates cellular and body changes during pregnancy remain unclear. Changes in these mechanisms can lead to preeclampsia, intrauterine growth restriction, rupture of membranes, and premature delivery. Therefore, the mechanisms of NO need to be further elucidated, and it is of great clinical significance to use NO to prevent these changes and treat fertility diseases.

## Author Contributions

CL and XZ designed the article. YL wrote the manuscript. YZ and WB contributed to manuscript writing. XW revised the manuscript. All authors contributed to the article and approved the submitted version.

## Funding

This work was supported by the National Natural Science Foundation of China (Grants Nos.32172726, 31772596) and the Open Project Program of State Key Laboratory of Hulless Barley and Yak. Germplasm Resources and Genetic Improvement (XZNKY-2021-C-014-K07). And the Key Research and Development Program of Jilin Province (20210202048NC, 20210202103NC).

## Conflict of Interest

WB and YZ are members of the State Key Laboratory of Barley Yak Germplasm Resources and Genetics, not the Lhasa LTD. Here are their detailed units: State Key Laboratory of Hulless Barley and Yak Germplasm Resources and Genetic Improvement, Lhasa, Tibet 850002, China.

The remaining authors declare that the research was conducted in the absence of any commercial or financial relationships that could be construed as a potential conflict of interest.

## Publisher’s Note

All claims expressed in this article are solely those of the authors and do not necessarily represent those of their affiliated organizations, or those of the publisher, the editors and the reviewers. Any product that may be evaluated in this article, or claim that may be made by its manufacturer, is not guaranteed or endorsed by the publisher.

## Abbreviation

NO, Nitric oxide; NOS, Nitric oxide synthase; nNOS, Neural NOS; eNOS, Endothelial NOS; iNOS, Inducible NOS; CNOS, Constituent (or structural) synthase; SGC, soluble guanylate cyclase; PKC, protein kinase C; GnRH, gonadotropin-releasing hormone; LH, Luteinizing Hormone; FSH, Follicle-Stimulating Hormone; SNP, Single nucleotide polymorphism.
